# Exome Sequencing of Uterine Leiomyosarcomas Identifies Frequent Mutations in *TP53*, *ATRX*, and *MED12*

**DOI:** 10.1371/journal.pgen.1005850

**Published:** 2016-02-18

**Authors:** Netta Mäkinen, Mervi Aavikko, Tuomas Heikkinen, Minna Taipale, Jussi Taipale, Riitta Koivisto-Korander, Ralf Bützow, Pia Vahteristo

**Affiliations:** 1 Genome-Scale Biology Research Program, Research Programs Unit, University of Helsinki, Helsinki, Finland; 2 Department of Medical and Clinical Genetics, Medicum, University of Helsinki, Helsinki, Finland; 3 Science for Life Laboratory, Department of Biosciences and Nutrition, Karolinska Institutet, Stockholm, Sweden; 4 Department of Obstetrics and Gynecology, Helsinki University Hospital and University of Helsinki, Helsinki, Finland; 5 Department of Pathology, The Laboratory of Helsinki University Hospital (HUSLAB), Helsinki University Hospital and Medicum, University of Helsinki, Helsinki, Finland; Brigham & Women's Hospital, UNITED STATES

## Abstract

Uterine leiomyosarcomas (ULMSs) are aggressive smooth muscle tumors associated with poor clinical outcome. Despite previous cytogenetic and molecular studies, their molecular background has remained elusive. To examine somatic variation in ULMS, we performed exome sequencing on 19 tumors. Altogether, 43 genes were mutated in at least two ULMSs. Most frequently mutated genes included *tumor protein P53* (*TP53*; 6/19; 33%), *alpha thalassemia/mental retardation syndrome X-linked* (*ATRX*; 5/19; 26%), and *mediator complex subunit 12* (*MED12*; 4/19; 21%). Unlike *ATRX* mutations, both *TP53* and *MED12* alterations have repeatedly been associated with ULMSs. All the observed *ATRX* alterations were either nonsense or frameshift mutations. ATRX protein levels were reliably analyzed by immunohistochemistry in altogether 44 ULMSs, and the majority of tumors (23/44; 52%) showed clearly reduced expression. Loss of ATRX expression has been associated with alternative lengthening of telomeres (ALT), and thus the telomere length was analyzed with telomere-specific fluorescence *in situ* hybridization. The ALT phenotype was confirmed in all ULMSs showing diminished ATRX expression. Exome data also revealed one nonsense mutation in *death-domain associated protein* (*DAXX*), another gene previously associated with ALT, and the tumor showed ALT positivity. In conclusion, exome sequencing revealed that *TP53*, *ATRX*, and *MED12* are frequently mutated in ULMSs. ALT phenotype was commonly seen in tumors, indicating that ATR inhibitors, which were recently suggested as possible new drugs for *ATRX*-deficient tumors, could provide a potential novel therapeutic option for ULMS.

## Author Summary

Uterine leiomyosarcomas are rare, malignant smooth muscle tumors with a poor 5-year survival and high recurrence rate. They account for 1–2% of all uterine malignancies with an estimated incidence of 0.4/100,000 women per year. The symptoms and signs of this tumor type widely overlap with those of common benign uterine leiomyomas, making early diagnosis of uterine leiomyosarcomas difficult. Currently, the diagnosis of these tumors is often incidental and postoperative. Despite previous cytogenetic and molecular studies, their molecular background has remained elusive. Identification of novel molecular genetic characteristics in uterine leiomyosarcomas is clinically relevant to further improve the diagnosis and prognosis of the patients. Here, we performed exome sequencing on 19 tumors, revealing frequent mutations in *TP53*, *ATRX*, and *MED12*. The discovery of frequent inactivating *ATRX* mutations provides a potential novel therapeutic target for uterine leiomyosarcomas.

## Introduction

Uterine leiomyosarcoma (ULMS) is a rare, highly malignant tumor that originates from the smooth muscle layer of the uterus, the myometrium. It is the most common subtype of uterine sarcoma and accounts for 1–2% of all uterine malignancies with an estimated incidence of 0.4/100,000 women per year [1,2]. The majority of ULMSs occur in women over 50 years of age typically causing symptoms such as abnormal vaginal bleeding, palpable pelvic mass, and abdominal pain. These symptoms greatly resemble those of common benign uterine leiomyoma, making early diagnosis of ULMS difficult. Surgical resection is the primary treatment option, while the use of adjuvant therapies varies widely. ULMS show low sensitivity to both chemotherapy and radiation therapy [3,4]. In most cases, the diagnosis is made histologically after the surgery, and even then, the clinical course of ULMS is difficult to predict. Currently, the most prominent prognostic factors include stage, age, and tumor size [5–7]. The 5-year overall survival has remained <50% due to a high recurrence rate (53–71%) and metastatic capacity [6,8].

Most ULMSs are aneuploid with both complex numerical and structural chromosomal aberrations [9]. Albeit no consistent structural aberrations have been identified, abnormalities affecting chromosomal regions 1p, 10q, 13q, and 14q have been observed in multiple cases [10]. So far, only a few genes have been associated with this tumor type, including *tumor protein P53* (*TP53*), *RB1*, *MDM2*, *CDKN2A*, and *KIT* [9,11]. These are all common cancer genes not specific for smooth muscle malignancies and the exact molecular mechanisms underlying ULMS tumorigenesis remain elusive.

During the last decade, next-generation sequencing technologies have increasingly provided genome-wide data on somatic landscapes in various cancer types enabling the discovery of novel cancer genes and mechanisms with important prognostic and therapeutic implications [12]. Here, we performed exome sequencing on 19 ULMSs to further elucidate the molecular etiology of these tumors, identifying frequent mutations in *TP53*, *alpha thalassemia/mental retardation syndrome X-linked* (*ATRX*), and *mediator complex subunit 12* (*MED12*). This is the first description of high-throughput sequencing on ULMSs.

## Results

### Recurrently mutated genes observed in exome sequencing

We performed exome sequencing on genomic DNA of 19 formalin-fixed paraffin-embedded (FFPE) ULMSs. The average coverage of captured exonic regions reached a mean depth of 21x and 92% of the captured regions had a minimum coverage of four reads (S1 Table). After filtering the exome sequencing data, we observed a mean of 373 somatic mutations per tumor (range 240–779). The majority of mutations in each tumor specimen represented single-nucleotide variations (∼88%; range 81–95%), while deletions accounted for ∼9% (range 4–15%) and insertions ∼3% (range 1–7%) (S1 Table). Two tumors, LMS49 and LMS51, harbored more mutations than other ULMSs, but the mutation spectrum followed the common pattern.

**Fig 1:**
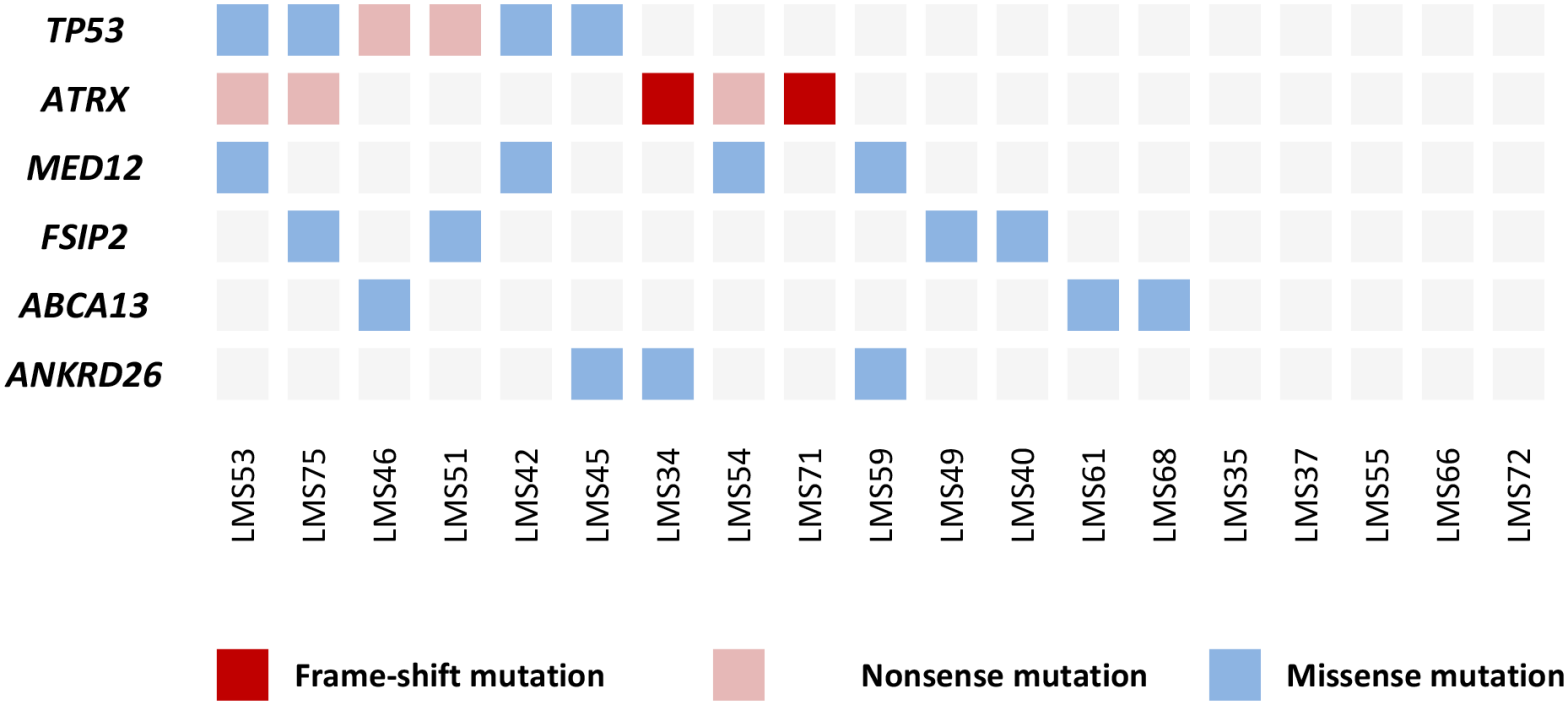
Most frequently mutated genes in 19 ULMSs studied by exome-sequencing. Five tumors did not have mutations in any of the six genes listed.

In the exome sequencing data analysis, we focused on genes that were mutated in at least two tumors. This resulted in a list of 43 genes (S2 Table). The majority of these genes (37/43; 86%) were mutated in two tumors, while six genes, *TP53*, *ATRX*, *MED12*, *fibrous sheath interacting protein 2* (*FSIP2)*, *ATP-binding cassette*, *sub-family A (ABC1)*, *member 13* (*ABCA13*), and *ankyrin repeat domain 26* (*ANKRD26*), had mutations in three or more tumors (Fig 1). The most frequently mutated gene was *TP53*, which was mutated in six tumors (6/19; 32%) (S1 Fig). Two mutations were nonsense mutations creating a premature stop-codon and four were missense alterations; all missense changes were predicted pathogenic by two independent *in silico* tools (S2 Table). All the observed *TP53* mutations have been reported as somatic mutations in the COSMIC-database.

The second most commonly mutated gene was *ATRX*, which was mutated in five tumors (5/19; 26%) (S1 Fig). The total number of mutations was six as one tumor (LMS71) contained two distinct mutations. All mutations were either nonsense mutations or small frameshift insertions or deletions, and were thus predicted to result in a truncated protein product. As *ATRX* mutations have been associated with alternative lengthening of telomeres (ALT), we specifically searched the exome sequencing data for possible mutations in *death-domain associated protein* (*DAXX*), as also these mutations have been associated with the ALT phenotype [13,14]. Indeed, one ULMS (LMS61) had a mutation in *DAXX*. This mutation was a nonsense mutation (Glu650Stop) most likely leading to a truncated or unstable protein product.

Four mutations (4/19; 21%) were observed in *MED12* (S1 Fig). All these were missense changes affecting amino acids Gly44 (3 mutations) or Leu36 (1 mutation), which have previously been reported as mutational hotspots in uterine leiomyomas [15]. All mutations were predicted to have a deleterious effect on protein function (S2 Table). Neither *MED12*, *TP53*, nor *ATRX* mutations were mutually exclusive (Fig 1).

Alterations in *FSIP2* (4/19; 21%), *ABCA13* (3/19; 16%), and *ANKRD26* (3/19; 16%) all represented missense changes that scattered along the gene lengths. Two tumors had the same Met487Ile substitution in *ANKRD26*. One alteration (Gln581Leu) in *FSIP2* and all changes in *ABCA13* were predicted pathogenic by both Polyphen-2 and SIFT, whereas none of the other variants were predicted damaging by both *in silico* tools.

### Aberrant TP53, ATRX, and DAXX expression in ULMS

**Fig 2:**
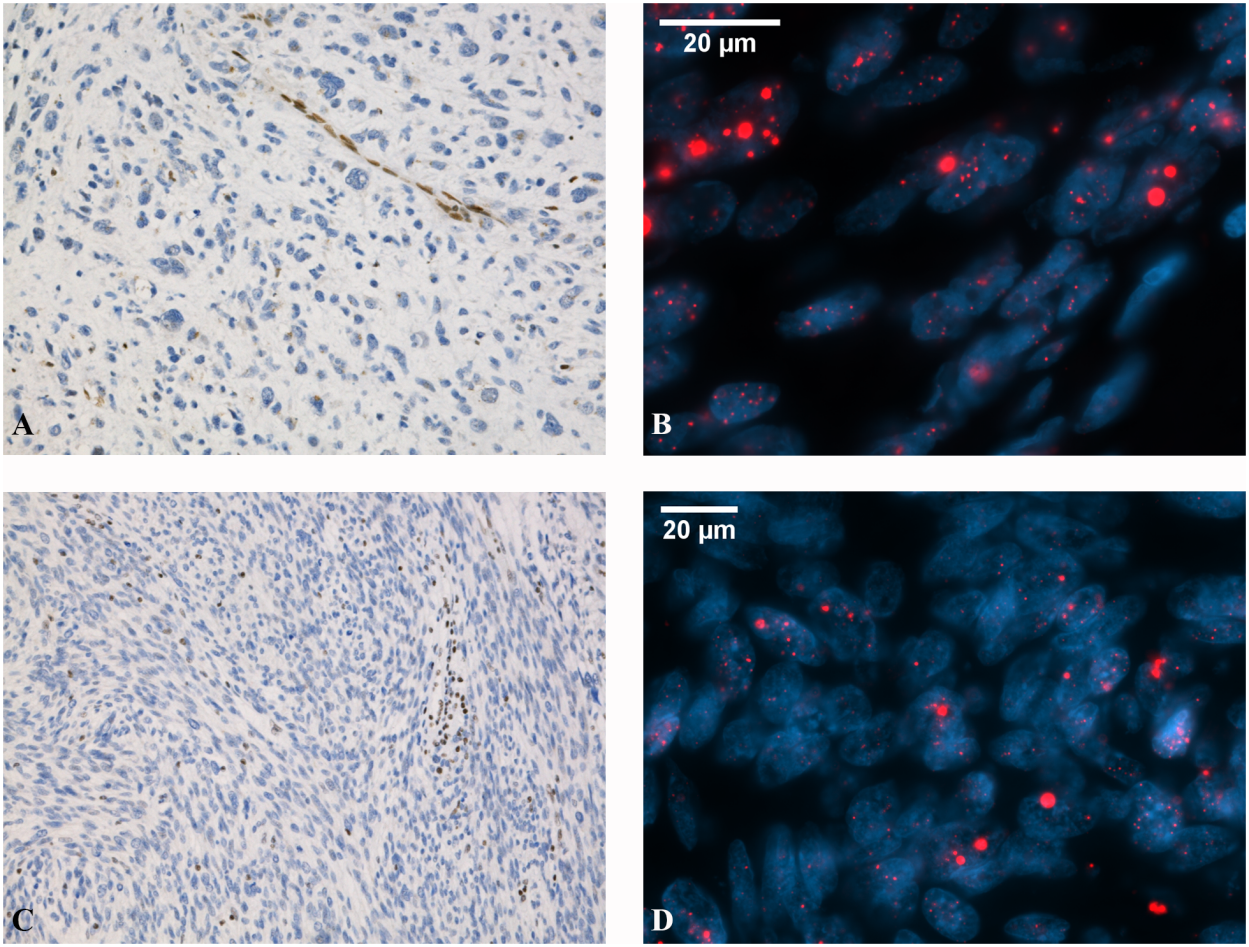
Representative images of ATRX and DAXX expression and ALT phenotype in ULMS. *ATRX*-mutated ULMS showing loss of ATRX expression **(A)** and positive ALT phenotype **(B)**. Large, abnormally bright telomere FISH signals (red) are indicative of ALT. *DAXX*-mutated ULMS with reduced DAXX expression **(C)** and positive ALT phenotype **(D)**. Immunohistochemical stainings are shown with 10×20 magnification and FISH stainings with 10×63 magnification.

We evaluated the protein expression levels of TP53, ATRX, and DAXX in the 19 exome-sequenced ULMSs by immunohistochemistry and validated the results in a larger set of 33 additional tumors (S2 Fig and S3 Table). DAXX immunostaining was successful in all 52 tumors and interpretable results for TP53 and ATRX were obtained from 50 and 44 tumors (50/52, 96%; 44/52, 85%). Aberrant TP53 expression was observed in 33 out of 50 ULMSs (66%) (S3 Table). Twenty-three out of 44 successfully analyzed ULMSs (52%) showed loss of nuclear ATRX expression, including all immunohistochemically successful *ATRX* mutation-positive tumors. Clearly diminished DAXX expression was present in only one ULMS (1/52, 2%) (Fig 2A and 2C): a tumor with the nonsense mutation (S3 Table).

### Fluorescence *in situ* hybridization shows alternative lengthening of telomeres

Telomere-specific fluorescence *in situ* hybridization (FISH) was carried out to assess the potential effect of *ATRX* and *DAXX* mutations on telomere length (Fig 2B and 2D). Twelve out of 19 exome-sequenced ULMSs (63%) were ALT-positive (S3 Table). This included four out of five *ATRX* mutation-positive tumors (80%) as well as the one *DAXX* mutation-positive tumor. Also seven out of 13 cases (54%) without detectable *ATRX* or *DAXX* mutations showed ALT positivity. Loss of ATRX or DAXX expression seems to correlate very well with the ALT phenotype.

### The effect of aberrant TP53 and ATRX expression on patient survival

**Fig 3:**
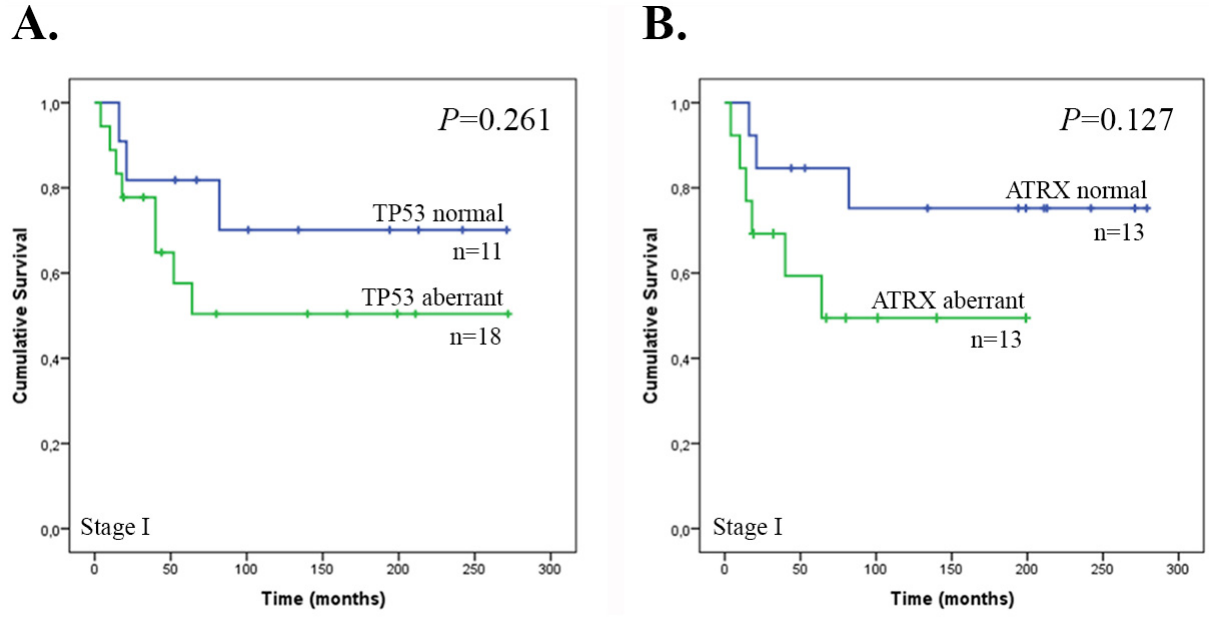
Overall survival of patients with Stage I ULMS according to TP53 and ATRX expression status. **(A)** TP53 expression (n = 29) and **(B)** ATRX expression (n = 26).

Kaplan-Meier survival curves were generated to study the association between TP53 and ATRX expression and overall survival time. The median overall survival time for all patients was 65 months (95% confidence interval 31.7–98.3 months). Only the number of Stage I tumors was large enough for the analyses. Neither aberrant TP53 or ATRX expression associated with poor survival (*P* = 0.261, *P* = 0.127) (Fig 3). Of note, TP53 and ATRX expression statuses correlated with each other (*P* = 0.005).

## Discussion

In this study, we examined somatic variation in 19 ULMSs by exome sequencing. We focused on genes, which were mutated in at least two tumors; altogether 43 such genes were identified. The most frequently mutated genes included *TP53*, *ATRX*, *MED12*, *FSIP2*, *ABCA13*, and *ANKRD26*. *TP53* was the most commonly mutated gene with 32% of the tumors harboring mutations. Alterations in *TP53* have been previously implicated in leiomyosarcomas and suggested to play a role in leiomyosarcoma pathogenesis [16–18]. In this study, most mutations (67%) were missense changes located in exons 4–8. This is in line with previous studies, where the majority of mutations have been missense mutations in exons 5–8, the most highly conserved region of the gene [9,17,19]. These mutations are known to alter the protein structure, inhibit its tumor suppressor function, and result in its prolonged half-life. Immunohistochemical analysis including 50 ULMSs confirmed altered expression in the majority of tumors, highlighting the role of *TP53* in ULMS development.

*ATRX* was the second most frequently mutated gene with mutations observed in five tumors (26%). All mutations were either nonsense or frameshift alterations most likely leading to a truncated protein product. Loss of ATRX expression has been reported in leiomyosarcomas of various sites [20–22] and a recent meeting abstract on ULMSs reported genomic alterations of this gene in 32% (8/25) of the studied tumors, supporting our findings [23]. We successfully analyzed ATRX protein levels in 44 ULMSs and showed that 52% of the tumors, including all reliably analyzed mutation-positive lesions, had clearly reduced expression. *ATRX* encodes a transcriptional regulator that contains an ATPase/helicase domain, and is thus a member of the SWI/SNF family of chromatin remodelling proteins. Loss of ATRX expression has been associated with ALT [13,24], which prompted us to analyze the telomeres with telomere-specific FISH. The ALT phenotype was confirmed in all ULMSs with diminished ATRX expression. Some exome-sequenced tumors with reduced ATRX expression and ALT positivity did not show *ATRX* mutations, suggesting that there are regulatory or larger structural alterations undetectable by exome sequencing, or that the quality of FFPE samples was inadequate to reveal the underlying mutation. Interestingly, the only ULMS with two *ATRX* mutations did not show ALT.

ATRX is known to functionally cooperate with DAXX and *DAXX* mutations have been associated with ALT [13,14]. We therefore scrutinized the exome data for possible *DAXX* mutations. One nonsense mutation was identified, and FISH confirmed the ALT phenotype. Overall, these results show that ALT is very common in ULMS and that in addition to *ATRX*, also *DAXX* mutations contribute to the phenotype. Importantly, ALT was recently suggested to render cancer cells hypersensitive to ATR inhibitors [25]. These inhibitors might provide a novel treatment for ULMS, in which chemotherapeutic options have thus far been limited.

*MED12* was mutated in four ULMSs (21%). All mutations were in exon 2, which is a known mutational hotspot in *MED12*. These mutations were first observed in uterine leiomyomas [15], and subsequently they have been identified in other tumor types [26]. Previous screening studies have reported recurrent *MED12* mutations also in ULMS with similar frequencies as observed here [26]. It may be that a subset of ULMSs arises through a leiomyoma precursor, or alternatively *MED12* mutations may provide growth advantage to ULMSs. MED12 is part of a multi-protein complex Mediator, which plays a key role in global transcription regulation in eukaryotic cells [27]. Based on our results, *MED12* mutations can co-occur with *TP53* and *ATRX* mutations.

*FSIP2*, *ABCA13*, and *ANKRD26* were mutated in at least three tumors and additional 37 genes had mutations in two tumors. Most alterations were missense changes and gave either neutral or controversial results in *in silico* predictions. The possible role of these genes in ULMS development cannot be directly assessed as in addition of providing growth advantage to the cell, the observed alterations may represent rare germline polymorphisms or passenger mutations with no functional significance.

Although aberrant expression of both TP53 and ATRX in Stage I ULMSs, the only group of tumors large enough for the analyses, did not associate with poor overall survival, a trend toward poorer survival was seen in the patients. The limited number of samples in the survival analyses and the observation that expression statuses were associated with each other makes it difficult to draw conclusions regarding prognostic implications of TP53 or ATRX expression levels. In general, *TP53* alterations are the most common genetic changes in human cancers and they are particularly associated with an aggressive phenotype. Recently, loss of ATRX expression was associated with poor clinical outcome in ULMS [21,22]. Larger sample series with information on both TP53 and ATRX are required to confirm these findings.

ULMSs are rare and aggressive cancers. In most cases the diagnosis is made only at surgery, and many patients thus present with an advanced disease. Here, we have utilized exome sequencing and identified several recurrently mutated genes, including *TP53*, *ATRX*, and *MED12*. While *MED12* mutations are the most common alterations in benign conventional leiomyomas, *TP53* or *ATRX* mutations have not been observed in these tumors. Specifically, identification of inactivating *ATRX* mutations and their association with the ALT phenotype in the substantial proportion of tumors may be translatable into clinical practice should the suggested effect of ATR inhibitors prove effective.

## Materials and Methods

### Ethics statement

This study was approved by the appropriate ethics review board of Hospital District of Helsinki and Uusimaa, Finland (408/13/03/03/2009).

### Patient samples

Fifty-two archival FFPE ULMS tissue samples were derived from the Department of Pathology, Hospital District of Helsinki and Uusimaa, Finland, according to Finnish laws and regulations by permission of the director of the respective health care unit. These specimens represented diagnostic ULMS samples collected during surgery in 1985–2013. Simultaneously with the sample collection, clinical data were obtained for these cases (Table 1) after which the samples were anonymized for the study. Nineteen ULMSs (diagnosis 2003–2013) entered exome sequencing, while the remaining 33 tumors were available on a tissue microarray for immunohistochemistry.

**Table 1:** Clinical data of 52 ULMSs.

Case ID	Age at diagnosis	Primary stage	Tumor size (cm)	Tumor necrosis	Mitotic index/10 HPF	Atypia (/3)	Metastasis	Patient status	Follow-up time (months)
**Exome-sequenced ULMSs**									
LMS34	81	IC	7	+	5	2	+	AWD	67
LMS35	63	IIB	>10	+	10	2	+	DOD	23
LMS37	67	IIIB	5	+	10	3	+	DOD	18
LMS40	56	IB	6	-	>20	2	-	NED	53
LMS42	66	N/A	15	+	50	2	+	DOD	18
LMS45	53	IA	3.5	-	>20	2	-	NED	32
LMS46	50	IC	12	+	>20	3	-	NED	14
LMS49	56	N/A	6.5	+	>20	3	+	DOD	41
LMS51	58	IB	30	+	N/A	3	+	AWD	44
LMS53	69	IC	15	+	16	3	-	alive	80
LMS54	76	N/A	6	-	>20	1	+	DOD	17
LMS55	56	N/A	1.5	+	>20	3	-	NED	97
LMS59	59	IVB	14	+	14	2	+	DOD	15
LMS61	54	I	N/A	+	33	2	+	DOD	16
LMS66	52	IB	7	+	30	2	-	NED	19
LMS68	48	N/A	>10	N/A	>10	3	+	DOD	90
LMS71	72	IC	11	-	>20	2	-	deceased^a^	40
LMS72	28	I	8	+	14	1	-	NED	101
LMS75	75	N/A	7	-	30	2	-	DOD	50
**ULMSs on tissue microarray**									
LMS1	69	IIIA	N/A	N/A	N/A	N/A	+	DOD	72
LMS2	44	IC	5	N/A	N/A	N/A	+	AWD	271
LMS3	52	IC	20	N/A	5	N/A	+	DOD	64
LMS4	72	IC	N/A	N/A	N/A	N/A	+	DOD	40
LMS5	41	IA	N/A	N/A	10	N/A	+	NED	279
LMS6	53	IB	12	N/A	N/A	N/A	-	NED	242
LMS7	77	IC	20	N/A	N/A	N/A	+	DOD	18
LMS8	77	IC	N/A	N/A	N/A	N/A	-	deceased^a^	82
LMS9	57	N/A	N/A	N/A	N/A	N/A	+	DOD	142
LMS10	42	III	5	N/A	8	N/A	+	NED	225
LMS11	58	I	4	N/A	N/A	N/A	-	NED	199
LMS12	71	IIIA	20	N/A	20	N/A	-	DOD	6
LMS13	48	IIIA	N/A	N/A	N/A	N/A	+	DOD	8
LMS14	64	IVB	5	N/A	N/A	N/A	-	DOD	5
LMS15	64	IIIA	8	N/A	16	N/A	+	DOD	65
LMS16	69	IIIA	N/A	N/A	N/A	N/A	-	DOD	122
LMS17	34	IC	4	N/A	10	N/A	-	NED	194
LMS18	64	IC	10	N/A	5	N/A	-	deceased^a^	21
LMS19	57	N/A	20	N/A	N/A	N/A	+	DOD	26
LMS20	81	IC	14	N/A	N/A	N/A	-	DOD	4
LMS21	59	IVA	N/A	N/A	N/A	N/A	+	DOD	14
LMS22	70	IC	N/A	N/A	N/A	N/A	+	DOD	10
LMS23	49	IC	10	N/A	N/A	N/A	-	NED	272
LMS24	49	IC	6	N/A	N/A	N/A	-	NED	166
LMS25	38	IC	4	N/A	N/A	N/A	-	NED	211
LMS27	62	IIB	10	N/A	10	N/A	+	DOD	61
LMS28	52	IC	N/A	N/A	20	N/A	-	NED	199
LMS29	42	IC	7	N/A	N/A	N/A	-	NED	213
LMS30	60	IIB	15	N/A	N/A	N/A	+	DOD	11
LMS31	40	IC	9	N/A	N/A	N/A	+	DOD	52
LMS57	81	IVB	15	+	>20	3	+	DOD	1
LMS62	56	IB	4	-	10	2	-	NED	140
LMS64	54	I	9	-	15	3	+	NED	134

^a^ cause of death other than ULMS

HPF, high power field; N/A, not available; DOD, died of disease; NED, no evidence of disease; AWD, alive with disease

### Histological evaluation

Before exome sequencing, hematoxylin-eosin-stained sections from each specimen were re-evaluated by a pathologist (RB) and verified as ULMSs according to the WHO criteria [28]. Tumor percentage was ≥90% in all samples.

### Exome sequencing

Genomic DNA was extracted with a standard phenol-chloroform method. Sample libraries were prepared using NEBNext DNA Library Prep Reagent Set for Illumina (New England Biolabs Ltd. catalog# E6000) and subjected to exome capture with NimbleGen SeqCap EZ System (Roche NimbleGen). Paired-end short read sequencing was performed with HiSeq 2000 (Illumina Inc.) at Karolinska Institutet, Sweden.

### Somatic variant calling

Read mapping and somatic variant calling were carried out as previously described [29]. Additionally, single duplicate reads were removed with an in-house script.

### Variant identification

Exome data was analyzed with an in-house analysis and visualization tool RikuRator. The requirements to call a variant included a minimum coverage of six reads and the mutated allele to be present in at least 20% of the reads. The Genome Analysis Toolkit (GATK) quality score of variants was required to be 25 or above. Both exonic regions and sequences within three base pairs of the exon-intron boundaries were included in the study. Synonymous changes, variants present in the dbSNP database (release 138), and variants in Exome Aggregation Consortium Server with a frequency over 0.1% were disregarded. To remove other potential germline variants, the exome data was filtered against data from 2315 Finnish controls (93 individuals from the 1000 Genomes Project, 1941 individuals from The Sequencing Initiative Suomi (SISu) (http://www.sisu.fimm.fi), and 281 in-house control exomes or genomes). Recently, it has been estimated that about 400 control samples remove germline variation (single-nucleotide variants and indels) from a tumor sample at least as efficiently as the matched normal sample [30]. Lastly, all the remaining variants were individually visualized with Rikurator to exclude those only present in the same direction reads as likely artifacts. The functional effects of the variants were predicted by two independent *in silico* tools: SIFT (http://sift.jcvi.org/) and Polyphen-2 (http://genetics.bwh.harvard.edu/pph2/).

### Direct sequencing

All candidate variants in genes mutated in at least three tumors were validated by direct sequencing. Oligonucleotide primers were designed with Primer3Plus software (http://www.bioinformatics.nl/cgi-bin/primer3plus/primer3plus.cgi) (S4 Table). PCR products were sequenced directly utilizing Big Dye Terminator v.3.1 sequencing chemistry (Applied Biosystems) on an ABI3730 Automatic DNA Sequencer.

### Immunohistochemistry

TP53, ATRX, and DAXX immunolabeling was performed on FFPE sections of all 19 exome-sequenced ULMSs and on a tissue microarray containing 33 additional tumors. For TP53, immunostaining was performed as previously described [31]. For ATRX and DAXX, heat-induced antigen retrieval was carried out in a microwave using citrate buffer (pH 6.0) for 20 min. Endogenous peroxidase blocking was followed by overnight incubation with the primary antibody at 4°C (anti-ATRX 1:500 dilution, Sigma-Aldrich catalog# HPA001906; anti-DAXX 1:500 dilution, Sigma-Aldrich catalog# HPA008736). The primary antibody was detected with DAB Plus Substrate System (Thermo Fisher Scientific catalog# TA-060-HDX). Immunohistochemical scoring was assessed by a pathologist (RB). Only nuclear labeling of the proteins was evaluated. The loss of nuclear staining in tumor cells together with retained expression in non-neoplastic cells (endothelial or inflammatory cells) was considered loss of expression. The scoring was done without knowledge of the clinical outcome data.

### Telomere-specific FISH and microscopy

Detection of large, abnormally intense, intra-nuclear telomere DNA aggregates via telomere-specific FISH is considered the most sensitive and specific marker for identifying ALT phenotype in fixed tissue samples [13].

FFPE sections were deparaffinized at room temperature with xylene (3x10 min) and 100% EtOH (2x10 min) and air-dried. Subsequently, the slides were rinsed in phosphate-buffered saline (PBS) at 37°C (2x5 min) followed by RNAse A treatment (Sigma-Aldrich catalog# R4642) at 37°C for an hour. After a series of washes at room temperature with saline-sodium citrate (pH 7.0; 3x5 min) and deionized water (2x5 min), the slides were digested with Digest All 3-pepsin (Invitrogen/Life Technologies catalog# 00–3009) at 37°C for 10 min and rinsed with PBS at room temperature (2x5 min). Next, the slides were dehydrated and hybridized with Cy3-labeled peptide nucleic acid (PNA) probe (Panagene Inc. catalog# F1006-5). The denaturation took place at 85°C for 10 min and hybridization in dark at room temperature for an hour. Post-hybridization washes with saline-sodium citrate/0.1% Tween-20 (pH 7.0; 2x10 min) at 55°C and at room temperature for 10 min were followed by nuclear counterstaining with DAPI. The slides were imaged with a Zeiss Axio Imager epifluorescence microscope and image acquisition took place through Hamamatsu Orca Flash 4.0 LT camera and Zen software. The assessment of FISH slides was carried out independently by two authors (NM, MA). ULMSs were classified as ALT-positive if ≥5% of 300 assessed neoplastic cells displayed ALT-associated, abnormally bright telomeric DNA aggregates. In all cases, regions of necrosis and overlapping cells difficult to interpret were excluded from consideration.

### Statistical analyses

Statistical analyses were performed using SPSS statistical software for Windows version 22.0 (SPSS Inc.). Here, survival was defined as overall survival time from the time of diagnosis. Survival curves related to TP53 and ATRX expression were generated using the Kaplan–Meier method, and median survival times with 95% confidence intervals were given. Comparison of survival curves between normal and aberrant expression was performed using the log-rank test. *P-*value <0.05 was considered statistically significant. Association between TP53 and ATRX expression statuses was evaluated using cross tabulation and Fisher’s exact test.

## Supporting Information

S1 Fig The types and positions of the observed *TP53*, *ATRX*, and *MED12* mutations. (PDF)

S2 Fig Representative images of TP53, ATRX, and DAXX immunohistochemistry, as well as ALT phenotype in ULMSs. For TP53, heterogeneous expression of the protein was considered normal, while overexpression or loss of nuclear staining in tumor cells compared to the expression of non-neoplastic cells was considered aberrant. For ATRX and DAXX, loss of nuclear staining in tumor cells together with retained expression in non-neoplastic cells was considered loss of expression. In the case of ALT, abnormally bright telomere FISH signals indicated positive ALT status. Immunohistochemical stainings are shown with 10×20 magnification and FISH stainings with 10×63 magnification.
(PDF)

S1 Table Landscape of somatic mutations in 19 exome-sequenced ULMSs. (PDF)

S2 Table Summary of all 43 genes mutated in at least two ULMSs in the exome sequencing data. (PDF)

S3 Table *TP53*, *ATRX*, and *DAXX* mutation statuses, immunohistochemistry of TP53, ATRX, and DAXX, and ALT phenotype in the studied ULMSs. (PDF)

S4 Table Primer sequences and PCR conditions for validation of the exome sequencing data. (PDF)
