# Supplementary figures and images for: Exome Sequencing of Uterine Leiomyosarcomas Identifies Frequent Mutations in TP53, ATRX, and MED12
Source: PLoS Genet. 2016 Feb 18;12(2):e1005850. doi: 10.1371/journal.pgen.1005850 (PMC4758603; doi:10.1371/journal.pgen.1005850)

**Supplementary Figure S1.** The types and positions of the observed *TP53*, *ATRX*, and *MED12* mutations.

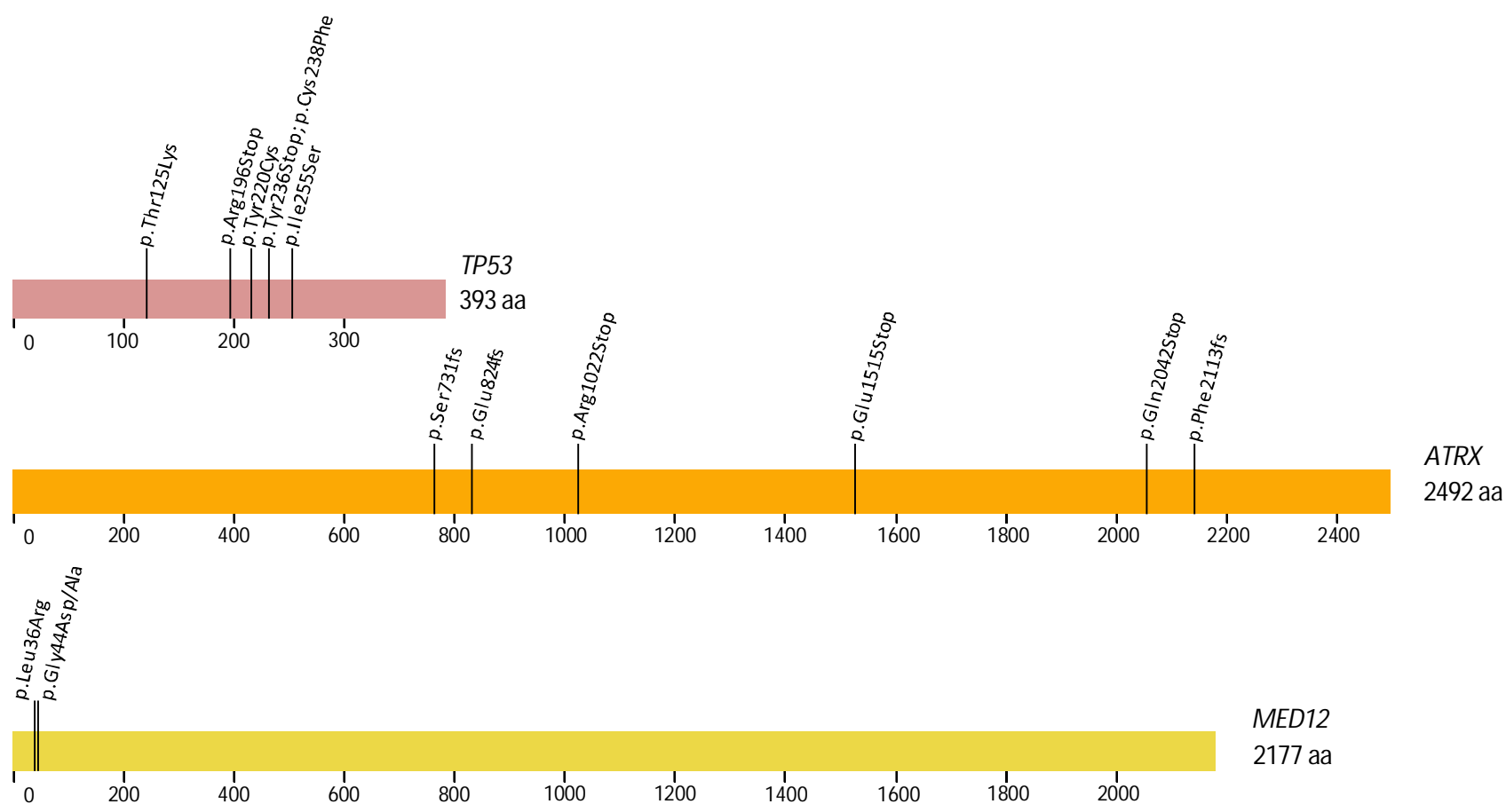

Supplement: S1 Fig — (PDF) [file pgen.1005850.s001.pdf]
