# Supplementary material for: Exome Sequencing of Uterine Leiomyosarcomas Identifies Frequent Mutations in TP53, ATRX, and MED12
Source: PLoS Genet. 2016 Feb 18;12(2):e1005850. doi: 10.1371/journal.pgen.1005850 (PMC4758603; doi:10.1371/journal.pgen.1005850)
Supplement: S2 Fig — For TP53, heterogeneous expression of the protein was considered normal, while overexpression or loss of nuclear staining in tumor cells compared to the expression of non-neoplastic cells was considered aberrant. For ATRX and DAXX, loss of nuclear staining in tumor cells together with retained expression in non-neoplastic cells was considered loss of expression. In the case of ALT, abnormally bright telomere FISH signals indicated positive ALT status. Immunohistochemical stainings are shown with 10×20 magnification and FISH stainings with 10×63 magnification. (PDF) [file pgen.1005850.s002.pdf]

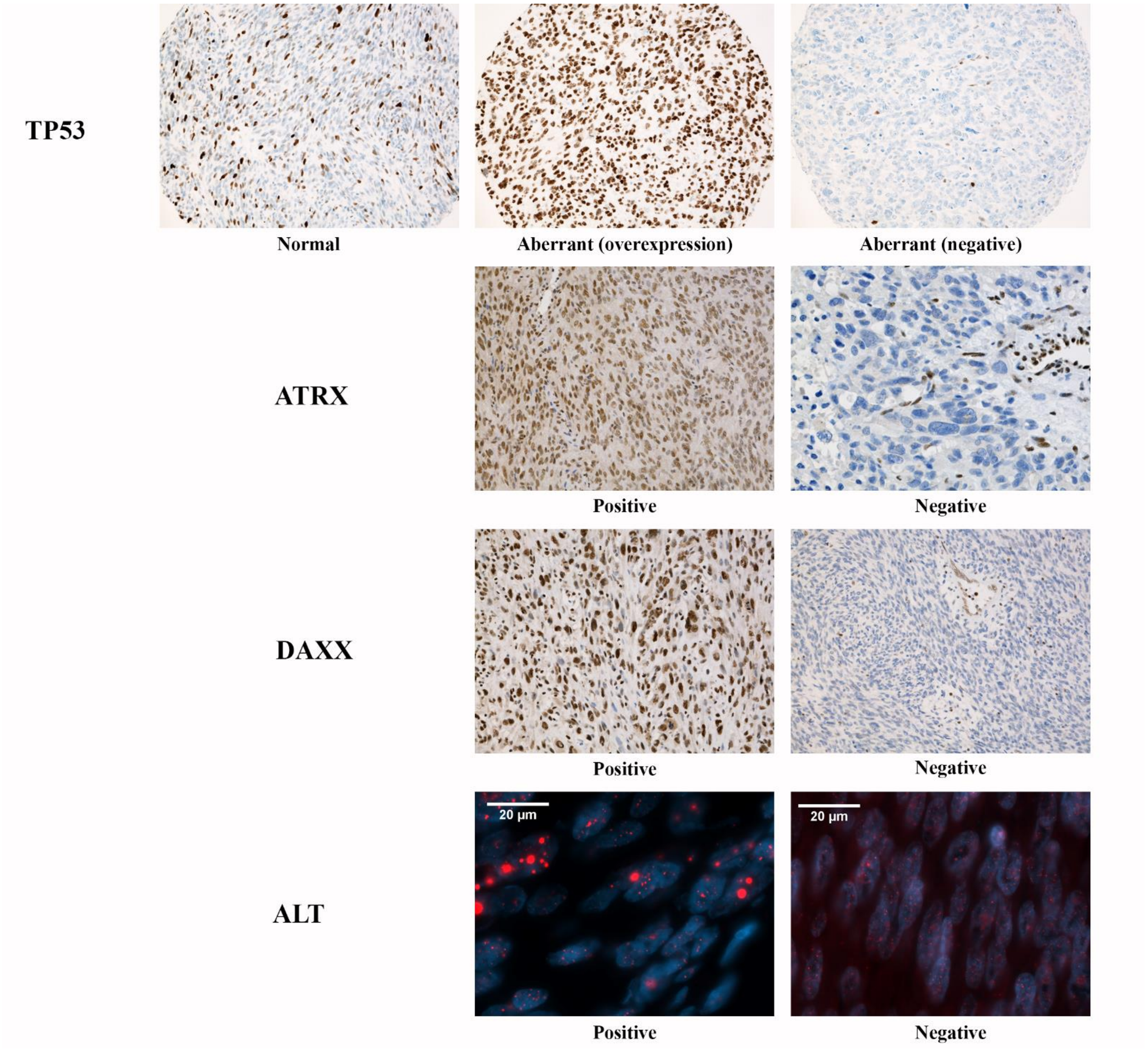

Supplementary Figure S2. Representative images of TP53, ATRX, and DAXX immunohistochemistry, as well as ALT phenotype in ULMSs. For TP53, heterogeneous expression of the protein was considered normal, while overexpression or loss of nuclear staining in tumor cells compared to the expression of non-neoplastic cells was considered aberrant. For ATRX and DAXX, loss of nuclear staining in tumor cells together with retained expression in non-neoplastic cells was considered loss of expression. In the case of ALT, abnormally bright telomere FISH signals indicated positive ALT status. Immunohistochemical stainings are shown with 10×20 magnification and FISH stainings with 10×63 magnification.
