# Supplementary material for: Exome Sequencing of Uterine Leiomyosarcomas Identifies Frequent Mutations in TP53, ATRX, and MED12
Source: PLoS Genet. 2016 Feb 18;12(2):e1005850. doi: 10.1371/journal.pgen.1005850 (PMC4758603; doi:10.1371/journal.pgen.1005850)
Supplement: S1 Table — (PDF) [file pgen.1005850.s003.pdf]

Supplementary Table S1. Landscape of somatic mutations in the 19 exome-sequenced ULMSs.

Case ID	Average coverage	Covered ≥ 4 reads (%)	Total variant count[a]	SNVs (%)	Deletions (%)	Insertions (%)	Mutations per Mb[b]
LMS34	20.52	91	293	247 (84)	38 (13)	8 (3)	5.25
LMS35	14.81	81	259	230 (89)	24 (9)	5 (2)	5.17
LMS37	20.68	96	317	288 (91)	19 (6)	10 (3)	5.43
LMS40	20.58	95	374	325 (87)	37 (10)	12 (3)	6.4
LMS42	27.99	96	408	356 (87)	39 (10)	13 (3)	6.99
LMS45	27.41	97	278	225 (81)	41 (15)	12 (4)	4.66
LMS46	25.89	91	349	302 (87)	35 (10)	12 (3)	6.63
LMS49	19.13	92	568	503 (89)	52 (9)	13 (2)	10.53
LMS51	23.27	95	779	737 (95)	35 (4)	7 (1)	13.64
LMS53	27.64	95	384	337 (88)	37 (10)	10 (2)	6.72
LMS54	19.72	90	369	324 (88)	31 (8)	14 (4)	7.37
LMS55	22.91	93	456	406 (89)	37 (8)	13 (3)	7.98
LMS59	20.48	93	277	231 (83)	28 (10)	18 (7)	4.9
LMS61	20.01	82	441	398 (90)	40 (9)	3 (1)	9.28
LMS66	17.81	94	301	268 (89)	24 (8)	9 (3)	5.21
LMS68	14.99	89	240	219 (91)	17 (7)	4 (2)	4.92
LMS71	18.51	94	338	298 (88)	27 (8)	13 (4)	5.85
LMS72	20.76	94	266	228 (86)	32 (12)	6 (2)	4.66
LMS75	19.82	93	403	371 (92)	24 (6)	8 (2)	7.47

[a]Total variant count comprises all those variants which were located into positions with a minimum coverage of six reads and the mutated allele present in at least 20% of the reads, as well as remained after filtering the sequencing data against 93 Finnish individuals from the 1000 Genomes Project, 1941 Finnish individuals from The Sequencing Initiative Suomi (SISu), and 281 in-house control exomes or -genomes.
[b]Mutations per Mb was calculated by dividing the total number of somatic mutations by the total number of called nucleotide positions (≥6 reads).

SNV, single-nucleotide variation
