# Supplementary material for: Exome Sequencing of Uterine Leiomyosarcomas Identifies Frequent Mutations in TP53, ATRX, and MED12
Source: PLoS Genet. 2016 Feb 18;12(2):e1005850. doi: 10.1371/journal.pgen.1005850 (PMC4758603; doi:10.1371/journal.pgen.1005850)
Supplement: S2 Table — (PDF) [file pgen.1005850.s004.pdf]

Supplementary Table S2. Summary of all 43 genes mutated in at least two ULMSs in the exome sequencing data.

Case ID	Gene	Ensembl gene	Ensembl transcript	Position	Direction	Exon	Nucleotide change	Amino acid change	Calls	Coverage	Calls/Coverage	Score	Polyphen-2	SIFT
LMS42	TP53	ENSG00000141510	ENST00000269305	17:7577517	-1	7	A->C	Ile255Ser	26	28	0.92	742.94	Probably damaging	Damaging
LMS45	TP53	ENSG00000141510	ENST00000269305	17:7578190	-1	6	T->C	Tyr220Cys	15	21	0.71	431.77	Probably damaging	Damaging
LMS46	TP53	ENSG00000141510	ENST00000269305	17:7577573	-1	7	G->C	Tyr236Stop	7	12	0.58	185.77	N/A	N/A
LMS51	TP53	ENSG00000141510	ENST00000269305	17:7578263	-1	6	G->A	Arg196Stop	8	8	1.0	226.8	N/A	N/A
LMS53	TP53	ENSG00000141510	ENST00000269305	17:7579313	-1	4	G->T	Thr125Lys	20	27	0.74	556.77	Probably damaging	Damaging
LMS75	TP53	ENSG00000141510	ENST00000269305	17:7577568	-1	7	C->A	Cys238Phe	13	17	0.76	332.77	Probably damaging	Damaging
LMS34	ATRX	ENSG00000085224	ENST00000373344	X:76814303	-1	29	DEL->ATAA	Fshift4	21	41	0.51	1144.73	N/A	N/A
LMS53	ATRX	ENSG00000085224	ENST00000373344	X:76937684	-1	9	G->A	Arg1022Stop	11	23	0.47	289.77	N/A	N/A
LMS54	ATRX	ENSG00000085224	ENST00000373344	X:76845397	-1	27	G->A	Gln2042Stop	10	24	0.41	220.77	N/A	N/A
LMS71	ATRX	ENSG00000085224	ENST00000373344	X:76938279	-1	9	INS->T	Fshift1	17	38	0.44	397.73	N/A	N/A
LMS71	ATRX	ENSG00000085224	ENST00000373344	X:76938556	-1	9	DEL->G	Fshift1	8	12	0.66	242.73	N/A	N/A
LMS75	ATRX	ENSG00000085224	ENST00000373344	X:76907618	-1	15	C->A	Glu1515Stop	18	25	0.72	482.77	N/A	N/A
LMS42	MED12	ENSG00000184634	ENST00000374080	X:70339230	1	2	T->G	Leu36Arg	9	28	0.32	168.77	Probably damaging	Damaging
LMS53	MED12	ENSG00000184634	ENST00000374080	X:70339254	1	2	G->A	Gly44Asp	10	13	0.76	267.77	Probably damaging	Damaging
LMS54	MED12	ENSG00000184634	ENST00000374080	X:70339254	1	2	G->C	Gly44Ala	6	7	0.85	172.9	Probably damaging	Damaging
LMS59	MED12	ENSG00000184634	ENST00000374080	X:70339254	1	2	G->A	Gly44Asp	3	9	0.33	47.77	Probably damaging	Damaging
LMS40	FSIP2	ENSG00000188738	ENST00000343098	2:186656875	1	16	A->G	Asn1760Ser	5	12	0.41	115.77	Benign	Tolerated
LMS49	FSIP2	ENSG00000188738	ENST00000343098	2:186661995	1	16	G->A	Glu3467Lys	6	16	0.37	117.77	Benign	Damaging
LMS51	FSIP2	ENSG00000188738	ENST00000343098	2:186650984	1	15	A->T	Gln581Leu	40	43	0.93	1253.82	Probably damaging	Damaging
LMS75	FSIP2	ENSG00000188738	ENST00000343098	2:186670433	1	17	G->A	Gly5556Asp	7	17	0.41	161.77	Benign	Damaging
LMS46	ABCA13	ENSG00000179869	ENST00000435803	7:48287957	1	14	G->C	Arg594Pro	6	9	0.66	128.77	Probably damaging	Damaging
LMS61	ABCA13	ENSG00000179869	ENST00000435803	7:48428817	1	37	T->C	Ile3885Thr	12	22	0.54	290.77	Possibly damaging	Damaging
LMS68	ABCA13	ENSG00000179869	ENST00000435803	7:48312200	1	17	G->C	Gln979His	12	13	0.92	309.89	Probably damaging	Damaging (low confidence)
LMS34	ANKRD26	ENSG00000107890	ENST00000376087	10:27324363	-1	24	C->T	Val1006Ile	25	41	0.60	680.77	Benign	Tolerated
LMS45	ANKRD26	ENSG00000107890	ENST00000376087	10:27350074	-1	13	C->G	Met487Ile	13	40	0.32	304.77	Possibly damaging	Tolerated
LMS59	ANKRD26	ENSG00000107890	ENST00000376087	10:27350074	-1	13	C->G	Met487Ile	14	24	0.58	339.77	Possibly damaging	Tolerated
LMS46	SYDE2	ENSG00000097096	ENST00000341460	1:85656402	-1	2	T->C	Lys260Arg	20	49	0.40	500.77	Probably damaging	Tolerated
LMS61	SYDE2	ENSG00000097096	ENST00000341460	1:85656402	-1	2	T->C	Lys260Arg	15	22	0.68	373.77	Probably damaging	Tolerated

Case ID	Gene	Ensembl gene	Ensembl transcript	Position	Direction	Exon	Nucleotide change	Amino acid change	Calls	Coverage	Calls/Coverage	Score	Polyphen-2	SIFT
LMS51	CACNA1E	ENSG00000198216	ENST00000367573	1:181741311	1	37	G->A	Gly1695Ser	7	25	0.28	116.77	Probably damaging	Damaging
LMS53	CACNA1E	ENSG00000198216	ENST00000367573	1:181480647	1	3	G->T	spl1	11	20	0.55	284.77	N/A	N/A
LMS51	NEB	ENSG00000183091	ENST00000397345	2:152423804	-1	114	C->T	Ala6012Thr	25	26	0.96	806.77	Probably damaging	Damaging
LMS54	NEB	ENSG00000183091	ENST00000397345	2:152381092	-1	152	G->T	Tyr7437Stop	3	8	0.37	28.77	N/A	N/A
LMS55	CCDC140	ENSG00000163081	ENST00000295226	2:223168970	1	2	C->A	Pro117Thr	5	7	0.71	109.77	Benign	N/A
LMS71	CCDC140	ENSG00000163081	ENST00000295226	2:223168899	1	2	C->A	Ala93Asp	7	14	0.5	126.77	Probably damaging	N/A
LMS72	HDLBP	ENSG00000115677	ENST00000391975	2:242181938	-1	17	G->C	Ile702Met	5	14	0.35	77.77	Probably damaging	Damaging
LMS75	HDLBP	ENSG00000115677	ENST00000391975	2:242194839	-1	8	G->A	Arg344Stop	3	14	0.21	34.77	N/A	N/A
LMS40	SETD5	ENSG00000168137	ENST00000402198	3:9495453	1	17	A->G	Met793Val	19	34	0.55	518.77	Possibly damaging	Tolerated
LMS72	SETD5	ENSG00000168137	ENST00000402198	3:9475584	1	4	C->T	His43Tyr	8	20	0.4	167.77	Probably damaging	Damaging
LMS35	KIAA2018	ENSG00000176542	ENST00000316407	3:113379379	-1	7	T->C	Ile384Val	8	12	0.66	174.77	Benign	Tolerated
LMS45	KIAA2018	ENSG00000176542	ENST00000316407	3:113376064	-1	7	A->G	Tyr1489His	11	22	0.5	295.77	Possibly damaging	Tolerated
LMS42	PLXNA1	ENSG00000114554	ENST00000393409	3:126723946	1	6	C->A	Ala586Asp	3	10	0.3	25.78	Benign	Damaging
LMS75	PLXNA1	ENSG00000114554	ENST00000393409	3:126724925	1	7	DEL->A	Fshift1	8	11	0.72	269.73	N/A	N/A
LMS72	WDFY3	ENSG00000163625	ENST00000295888	4:85701416	-1	26	T->C	Thr1404Ala	13	24	0.54	318.77	Benign	Tolerated
LMS75	WDFY3	ENSG00000163625	ENST00000295888	4:85672772	-1	36	T->C	Asn1946Ser	23	44	0.52	673.77	Benign	Tolerated
LMS49	TRPC7	ENSG00000069018	ENST00000513104	5:135692339	-1	2	C->T	Ser246Asn	4	19	0.21	53.77	Probably damaging	Damaging
LMS51	TRPC7	ENSG00000069018	ENST00000513104	5:135692463	-1	2	C->T	Glu205Lys	13	18	0.72	329.77	Benign	Tolerated
LMS51	TRPC7	ENSG00000069018	ENST00000513104	5:135692949	-1	2	C->T	Glu43Lys	8	18	0.44	186.77	Benign	Tolerated
LMS49	COL21A1	ENSG00000124749	ENST00000244728	6:56035780	-1	4	C->A	Val263Phe	16	51	0.31	346.77	Probably damaging	Tolerated
LMS55	COL21A1	ENSG00000124749	ENST00000244728	6:56035876	-1	4	A->G	Phe231Leu	15	29	0.51	408.77	Probably damaging	Damaging
LMS51	SHPRH	ENSG00000146414	ENST00000367505	6:146264655	-1	9	G->C	Ser621Cys	11	44	0.25	216.77	Possibly damaging	Tolerated
LMS61	SHPRH	ENSG00000146414	ENST00000367505	6:146262850	-1	10	C->T	Arg800His	12	35	0.34	269.77	Probably damaging	Damaging
LMS35	ADAM22	ENSG00000008277	ENST00000265727	7:87737525	1	5	G->C	Arg142Pro	9	28	0.32	206.77	Probably damaging	Damaging
LMS59	ADAM22	ENSG00000008277	ENST00000265727	7:87780626	1	20	T->C	Trp558Arg	11	23	0.47	220.77	Probably damaging	Damaging
LMS53	AKAP9	ENSG00000127914	ENST00000356239	7:91622340	1	5	G->A	Val183Ile	23	34	0.67	596.77	Benign	Tolerated
LMS68	AKAP9	ENSG00000127914	ENST00000356239	7:91672003	1	20	G->A	Glu1694Lys	13	29	0.44	327.77	N/A	Damaging
LMS55	CADPS2	ENSG00000081803	ENST00000449022	7:121965617	-1	29	T->A	Met1211Leu	11	19	0.57	265.77	Benign	Tolerated
LMS75	CADPS2	ENSG00000081803	ENST00000449022	7:122221279	-1	7	G->A	Thr430Ile	7	14	0.5	137.77	Possibly damaging	Damaging
LMS45	TRPV5	ENSG00000127412	ENST00000265310	7:142609805	-1	13	G->A	Pro544Leu	15	49	0.30	353.77	Probably damaging	Tolerated
LMS49	TRPV5	ENSG00000127412	ENST00000265310	7:142626553	-1	4	G->A	Arg153Cys	7	12	0.58	180.77	Probably damaging	Tolerated

Case ID	Gene	Ensembl gene	Ensembl transcript	Position	Direction	Exon	Nucleotide change	Amino acid change	Calls	Coverage	Calls/Coverage	Score	Polyphen-2	SIFT
LMS40	DLGAP2	ENSG00000198010	ENST00000421627	8:1616590	1	6	G->A	Glu556Lys	12	15	0.8	312.77	N/A	Damaging
LMS51	DLGAP2	ENSG00000198010	ENST00000421627	8:1497559	1	2	G->A	Val234Met	6	12	0.5	144.77	N/A	Tolerated
LMS51	CSMD1	ENSG00000183117	ENST00000537824	8:3015448	-1	39	C->T	Arg1962His	11	34	0.32	234.77	Probably damaging	Tolerated
LMS75	CSMD1	ENSG00000183117	ENST00000537824	8:2796181	-1	70	T->C	Thr3541Ala	7	17	0.41	170.77	Benign	Tolerated
LMS40	FZD3	ENSG00000104290	ENST00000240093	8:28384888	1	5	A->G	Tyr204Cys	11	31	0.35	271.77	Probably damaging	Damaging
LMS66	FZD3	ENSG00000104290	ENST00000240093	8:28385525	1	5	G->C	Lys416Asn	7	19	0.36	167.77	Probably damaging	Damaging
LMS40	TRPM6	ENSG00000119121	ENST00000360774	9:77390808	-1	24	C->T	Val1132Ile	19	40	0.47	505.77	Possibly damaging	Tolerated
LMS51	TRPM6	ENSG00000119121	ENST00000360774	9:77390940	-1	24	G->A	Arg1088Cys	10	19	0.52	232.77	Possibly damaging	Damaging
LMS49	GOLM1	ENSG00000135052	ENST00000388712	9:88642758	-1	10	G->A	Arg394Cys	9	25	0.36	175.77	Possibly damaging	Tolerated
LMS54	GOLM1	ENSG00000135052	ENST00000388712	9:88694199	-1	2	A->T	Ser13Thr	9	22	0.40	200.77	Probably damaging	Tolerated
LMS53	ZNF189	ENSG00000136870	ENST00000339664	9:104162190	1	2	G->C	Ala20Pro	29	52	0.55	849.77	Benign	Damaging
LMS68	ZNF189	ENSG00000136870	ENST00000339664	9:104170886	1	3	DEL->AAAG	Fshift4	16	23	0.69	932.73	N/A	N/A
LMS34	ANK3	ENSG00000151150	ENST00000280772	10:61827716	-1	38	T->G	Asp4099Ala	9	31	0.29	177.77	Probably damaging	Damaging
LMS34	ANK3	ENSG00000151150	ENST00000280772	10:61827717	-1	38	C->G	Asp4099His	9	31	0.29	174.77	Probably damaging	Damaging
LMS45	ANK3	ENSG00000151150	ENST00000280772	10:61844421	-1	32	A->G	Val1338Ala	15	27	0.55	386.77	Probably damaging	Damaging
LMS53	EIF3A	ENSG00000107581	ENST00000369144	10:120796639	-1	21	C->T	Arg1304His	34	56	0.60	928.77	Possibly damaging	Tolerated
LMS71	EIF3A	ENSG00000107581	ENST00000369144	10:120830448	-1	5	C->A	Glu231Stop	16	37	0.43	346.77	N/A	N/A
LMS45	OR5T2	ENSG00000181718	ENST00000313264	11:56000018	-1	1	C->T	Arg215His	19	52	0.36	454.77	Benign	Tolerated
LMS49	OR5T2	ENSG00000181718	ENST00000313264	11:55999635	-1	1	INS->T	Fshift1	6	14	0.42	108.73	N/A	N/A
LMS54	UTP20	ENSG00000120800	ENST00000261637	12:101705510	1	20	G->A	Glu749Lys	3	14	0.21	34.77	Possibly damaging	Tolerated
LMS72	UTP20	ENSG00000120800	ENST00000261637	12:101720867	1	26	A->G	Tyr1017Cys	9	19	0.47	232.77	Probably damaging	Damaging
LMS42	DNAH10	ENSG00000197653	ENST00000409039	12:124272362	1	10	C->A	Ala417Glu	5	13	0.38	96.77	Benign	Tolerated
LMS59	DNAH10	ENSG00000197653	ENST00000409039	12:124414259	1	71	C->T	Arg4071Trp	6	11	0.54	149.77	Probably damaging	Damaging
LMS34	PELI2	ENSG00000139946	ENST00000267460	14:56755267	1	4	G->T	Cys141Phe	8	13	0.61	186.77	Probably damaging	Damaging
LMS49	PELI2	ENSG00000139946	ENST00000267460	14:56755302	1	4	G->T	Ala153Ser	15	29	0.51	377.77	Probably damaging	Tolerated
LMS45	TMEM62	ENSG00000137842	ENST00000260403	15:43461854	1	11	C->T	Arg454Stop	11	18	0.61	296.77	N/A	N/A
LMS53	TMEM62	ENSG00000137842	ENST00000260403	15:43452909	1	10	C->T	His405Tyr	14	24	0.58	334.77	Probably damaging	Tolerated
LMS49	MAN2A2	ENSG00000196547	ENST00000360468	15:91463006	1	22	C->T	Arg1148Cys	15	26	0.57	420.77	Probably damaging	Damaging
LMS53	MAN2A2	ENSG00000196547	ENST00000360468	15:91454437	1	12	C->A	Arg638Ser	3	13	0.23	33.77	Benign	Damaging
LMS42	MYH11	ENSG00000133392	ENST00000396324	16:15932048	-1	2	A->G	Ile21Thr	15	37	0.40	340.77	Benign	Tolerated
LMS51	MYH11	ENSG00000133392	ENST00000396324	16:15847310	-1	16	T->C	Asn609Ser	10	25	0.4	210.77	Benign	Tolerated
LMS37	PHKB	ENSG00000102893	ENST00000323584	16:47727354	1	28	G->T	Arg944Leu	3	9	0.33	25.78	Benign	Tolerated
LMS51	PHKB	ENSG00000102893	ENST00000323584	16:47644808	1	14	G->A	Val479Met	16	50	0.32	361.77	Benign	Tolerated

Case ID	Gene	Ensembl gene	Ensembl transcript	Position	Direction	Exon	Nucleotide change	Amino acid change	Calls	Coverage	Calls/Coverage	Score	Polyphen-2	SIFT
LMS35	SPDYE4	ENSG00000183318	ENST00000328794	17:8660706	-1	2	C->T	Glu72Lys	7	17	0.41	149.77	Benign	Tolerated
LMS40	SPDYE4	ENSG00000183318	ENST00000328794	17:8656700	-1	5	C->T	Arg198Gln	4	12	0.33	50.77	Probably damaging	Tolerated
LMS55	GRIN2C	ENSG00000161509	ENST00000293190	17:72846379	-1	6	C->T	Arg486Gln	4	13	0.30	30.77	Possibly damaging	Tolerated
LMS71	GRIN2C	ENSG00000161509	ENST00000293190	17:72842266	-1	11	C->T	Met763Ile	9	12	0.75	223.77	Benign	Tolerated
LMS51	CNDP2	ENSG00000133313	ENST00000324262	18:72168570	1	3	G->A	Ala23Thr	8	36	0.22	122.77	Possibly damaging	Tolerated
LMS55	CNDP2	ENSG00000133313	ENST00000324262	18:72173148	1	4	DEL->AGA	Inframe3	6	13	0.46	227.73	N/A	N/A
LMS51	ASXL1	ENSG00000171456	ENST00000375687	20:31024474	1	13	C->T	Ala1320Val	4	13	0.30	75.77	Benign	Tolerated
LMS53	ASXL1	ENSG00000171456	ENST00000375687	20:31020711	1	11	G->T	Arg336Ser	12	22	0.54	292.77	Probably damaging	Damaging
LMS37	F8	ENSG00000185010	ENST00000360256	X:154158944	-1	14	T->A	Ile1041Phe	5	23	0.21	73.77	Benign	Tolerated
LMS45	F8	ENSG00000185010	ENST00000360256	X:154176114	-1	13	T->G	Ile658Leu	11	13	0.84	326.77	Probably damaging	Tolerated

N/A, not applicable
