# Supplementary material for: Exome Sequencing of Uterine Leiomyosarcomas Identifies Frequent Mutations in TP53, ATRX, and MED12
Source: PLoS Genet. 2016 Feb 18;12(2):e1005850. doi: 10.1371/journal.pgen.1005850 (PMC4758603; doi:10.1371/journal.pgen.1005850)
Supplement: S3 Table — (PDF) [file pgen.1005850.s005.pdf]

Supplementary Table S3. *TP53*, *ATRX*, and *DAXX* mutation statuses, immunohistochemistry of TP53, ATRX, and DAXX, and ALT phenotype in the studied ULMSs.

Case ID	*TP53/ATRX/DAXX* mutation status[a]	TP53 IHC[b]	ATRX IHC[c]	DAXX IHC[c]	ALT status
Exome-sequenced ULMSs					
LMS34	*ATRX* Pos	Normal	Neg	Pos	Pos
LMS35	Neg	Normal	Pos	Pos	Neg
LMS37	Neg	Aberrant (overexpression)	Neg	Pos	Pos
LMS40	Neg	Normal	Pos	Pos	Neg
LMS42	*TP53* Pos	Aberrant (overexpression)	Neg	Pos	Pos
LMS45	*TP53* Pos	Aberrant (overexpression)	Neg	Pos	Pos
LMS46	*TP53* Pos	Aberrant (negative)	Neg	Pos	Pos
LMS49	Neg	Normal	-	Pos	Neg
LMS51	*TP53* Pos	Aberrant (negative)	Pos	Pos	Neg
LMS53	*TP53/ATRX* Pos	Aberrant (overexpression)	Neg	Pos	Pos
LMS54	*ATRX* Pos	Aberrant (overexpression)	Neg	Pos	Pos
LMS55	Neg	Normal	Pos	Pos	Neg
LMS59	Neg	Normal	Pos	Pos	Neg
LMS61	*DAXX* Pos	Normal	Pos	Neg	Pos
LMS66	Neg	Aberrant (negative)	Neg	Pos	Pos
LMS68	Neg	Aberrant (negative)	-	Pos	Pos
LMS71	*ATRX* Pos	Aberrant (negative)	-	Pos	Neg
LMS72	Neg	Normal	Neg	Pos	Pos
LMS75	*TP53/ATRX* Pos	Aberrant (overexpression)	Neg	Pos	Pos
Additional ULMSs on tissue microarray					
LMS1	-	Aberrant (overexpression)	Pos	Pos	-
LMS2	-	Normal	Pos	Pos	-
LMS3	-	Aberrant (negative)	Neg	Pos	-
LMS4	-	Aberrant (overexpression)	Neg	Pos	-
LMS5	-	-	Pos	Pos	-

Case ID	*TP53/ATRX/DAXX* mutation status[a]	TP53 IHC[b]	ATRX IHC[c]	DAXX IHC[c]	ALT status
Additional ULMSs on tissue microarray					
LMS6	-	Normal	Pos	Pos	-
LMS7	-	Aberrant (negative)	Neg	Pos	-
LMS8	-	Normal	Pos	Pos	-
LMS9	-	Aberrant (negative)	Pos	Pos	-
LMS10	-	Normal	Pos	Pos	-
LMS11	-	Aberrant (negative)	Neg	Pos	-
LMS12	-	Aberrant (negative)	Pos	Pos	-
LMS13	-	Aberrant (overexpression)	Neg	Pos	-
LMS14	-	Aberrant (overexpression)	Neg	Pos	-
LMS15	-	Aberrant (overexpression)	Neg	Pos	-
LMS16	-	Aberrant (negative)	-	Pos	-
LMS17	-	Normal	Pos	Pos	-
LMS18	-	Normal	Pos	Pos	-
LMS19	-	Aberrant (overexpression)	Pos	Pos	-
LMS20	-	Aberrant (overexpression)	Neg	Pos	-
LMS21	-	Aberrant (overexpression)	-	Pos	-
LMS22	-	Aberrant (negative)	Neg	Pos	-
LMS23	-	Aberrant (negative)	-	Pos	-
LMS24	-	Aberrant (negative)	-	Pos	-
LMS25	-	Aberrant (negative)	Pos	Pos	-
LMS27	-	Normal	Neg	Pos	-
LMS28	-	Aberrant (overexpression)	Pos	Pos	-
LMS29	-	Normal	Pos	Pos	-
LMS30	-	Aberrant (overexpression)	Neg	Pos	-
LMS31	-	Aberrant (negative)	-	Pos	-

Case ID	*TP53/ATRX/DAXX* mutation status[a]	TP53 IHC[b]	ATRX IHC[c]	DAXX IHC[c]	ALT status
Additional ULMSs on tissue microarray					
LMS57	-	-	Neg	Pos	-
LMS62	-	Aberrant (overexpression)	Neg	Pos	-
LMS64	-	Normal	Pos	Pos	-

[a] The mutation status is based on exome sequencing data.

[b] TP53 immunohistochemistry (IHC) was scored either as aberrant (overexpression/loss of expression) or normal (heterogeneous expression).
Some results were ambiguous due to the malfunction of internal control cells (non-neoplastic cells).

[c] ATRX and DAXX IHC was scored as uniformly positive or negative.
Some results were ambiguous due to the malfunction of internal control cells (non-neoplastic cells).
