# Supplementary material for: Exome Sequencing of Uterine Leiomyosarcomas Identifies Frequent Mutations in TP53, ATRX, and MED12
Source: PLoS Genet. 2016 Feb 18;12(2):e1005850. doi: 10.1371/journal.pgen.1005850 (PMC4758603; doi:10.1371/journal.pgen.1005850)
Supplement: S4 Table — (PDF) [file pgen.1005850.s006.pdf]

Supplementary Table S4. Primer sequences and PCR conditions for validation of the exome sequencing data.

Gene	Exon	Alteration	Forward/Reverse	Sequence 5'-3'	Annealing temperature (°C)	Additional information
TP53 (ENST00000269305)	4	c.374C>A, p.Thr125Lys	Forward	CCCTTCCCAGAAAACCTACC	60	
			Reverse	GCCAAAGGGTGAAGAGGAAT		
	6	c.586C>T, p.Arg196Stop; c.659A>G, p.Tyr220Cys	Forward	GAGAGACGACAGGGCTGGT	60	
			Reverse	CACTGACAACCACCCTTAACC		
	7	c.708C>G, p.Tyr236Stop; c.713G>T, p.Cys238Phe; c.764T>G, p.Ile255Ser	Forward	CCACAGGTCTCCCCAAGG	60	
			Reverse	AGCAGGCCAGTGTGCAG		
ATRX (ENST00000373344)	9	c.2192delC, p.Ser731fs	Forward	AAGCGTAATTCTTCTGACAGTGC	60	
			Reverse	TGAAGAAGAACTGTGACTCATCC		
	9	c.2469insA, p.Glu824fs	Forward	AAGGGCAAATCAGCTAAGAGC	60	
			Reverse	TTTTGCTGTGTTTCTCATCTTCA		
	9	c.3064C>T, p.Arg1022Stop	Forward	AAGCAGAGCAAAAAGGGAACT	60	
			Reverse	CTTTTCTCCATCAGTTGTTCCA		
	15	c.4543G>T, p.Glu1515Stop	Forward	TCCTGGAAAAGGCAGAAAGA	60	
			Reverse	GCAACAGAGCAAGACCCTGTA		
	27	c.6124C>T, p.Gln2042Stop	Forward	TTTGTAGATCGATTTAGGAAGAAGTG	60	
			Reverse	GGGGTTTATCTTTATCTTCTGTCTTC		
	29	c.6338_6341delTTAT, p.Phe2113fs	Forward	AAGAAATGAATTCTCTGAACTCTTGA	60	
			Reverse	TCATAAGATGGATTCCAAGAAGC		
FSIP2 (ENST00000343098)	15	c.1742A>T, p.Gln581Leu	Forward	TCAATAATTAGAAATTTTGGGAAAAG	60	1.5 mM $MgCl_2$/25 µl
			Reverse	CATCAGAAAGTATGACTAATATGTTGC		
	16	c.5279A>G, p.Asn1760Ser	Forward	TGCATGCAAAGAAGGTATCAA	60	1.5 mM $MgCl_2$/25 µl
			Reverse	ATGCCTCCCCCTTCAGATT		
	16	c.10399G>A, p.Glu3467Lys	Forward	CAGCTGTGGCTTTCCAAGTC	60	
			Reverse	GCTGGCTTCAAGGTTTTCAT		
	17	c.16664G>A, p.Gly5556Asp	Forward	GCAGATGTGCAAAAGAGAACC	60	1.5 mM $MgCl_2$/25 µl
			Reverse	TTTTTCACTGATGTGTCCTTGG		
ABCA13 (ENST00000435803)	14	c.1781G>C, p.Arg594Pro	Forward	GAATATTTGGACTGGCAGGAA	60	1.5 mM $MgCl_2$/25 µl
			Reverse	CATTCTGATGTCCCAATTGTTT		
	17	c.2937G>C, p.Gln979His	Forward	CCTAAATGTCTTCCAGGACAAG	60	
			Reverse	CCCCAARAACCTCTGCTGT		
	37	c.11654T>C, p.Ile3885Thr	Forward	AGGGAAGGAGAGCTTGAAGG	60	
			Reverse	TTTTAAGAATCAAATGCCCAGT		
ANKRD26 (ENST00000376087)	13	c.1461G>C, p.Met487Ile	Forward	CTTCTTGCATGAGTGGATCA	60	
			Reverse	TGTTCTGAATTGATCAGCTTGG		
	24	c.3016G>A, p.Val1006Ile	Forward	TGGACGGCTTAGTGTTCTGA	60	
			Reverse	GCTCTCTGGAAARCAAGTTCTAGT		
DAXX (ENST00000374542)	7	c.1948G>T, p.Glu650Stop	Forward	CCTCCAGCCTCAGTCTTCC	60	
			Reverse	GGGAGAGTCCACCCTCGT		
